# Adrenocortical Activity and Aggressive Behavior in Children: A Longitudinal Study on Risk and Protective Effects

**DOI:** 10.3389/fpsyg.2021.636501

**Published:** 2021-04-22

**Authors:** Doris Bender, Friedrich Lösel

**Affiliations:** ^1^Institute of Psychology, University of Erlangen-Nürnberg, Erlangen, Germany; ^2^Institute of Criminology, University of Cambridge, Cambridge, United Kingdom

**Keywords:** adrenocortical activity, aggressive behavior, anxiousness, family stress, protective factors, pattern analysis

## Abstract

Most research on aggression and delinquency concentrates on risk factors. There has been less attention for protective factors and mechanisms, in particular with regard to biosocial influences. Based on theories of autonomous arousal and stress reactance the present study addresses the influence of adrenocortical activity as a risk and/or protective factor in the development of antisocial behavior in children. We also investigated relations to anxiousness and family stressors. In a prospective longitudinal study of 150 German boys, the first measurement took place at preschool age and contained an assessment of cortisol after waking up and 30 min later. Aggressiveness and anxiousness of the children were assessed by the kindergarten teachers with the Social Behavior Questionnaire. After 6 years, the children's behavior was rated by the teachers in middle school. Variable-oriented data analyses revealed a significant correlation between the total amount of cortisol after waking up and 30 min later (*AUC*_*G*_) and anxiousness both cross-sectionally and longitudinally, but not with aggressiveness. A family stress index correlated positively with aggressiveness but neither with cortisol nor with anxiousness. There were significant correlations between aggressiveness and anxiousness at kindergarten age and the respective behavior problems 6 years later. In a linear regression analysis on aggression only family stress had a significant effect but anxiousness not. Moderator analyses on aggressiveness with anxiousness and *AUC*_*G*_ or on *AUC*_*G*_ with anxiousness and aggressiveness did not show any significant interactions. Longitudinally, only aggression significantly predicted aggression 6 years later in a linear regression. In addition to variable-oriented analyses, we also applied a person-oriented approach to investigate specific patterns of behavior. Children who were high in both aggressiveness and anxiousness had the highest cortisol level and those with low anxiousness and high aggressiveness the lowest. The groups with different patterns of externalizing and internalizing problems at preschool age showed significant differences in aggression 6 years later. Our results underline the need for complex pattern analyses on cortisol, aggression, and anxiousness in children and for a differentiated consideration of emotional reactive aggression and unemotional instrumental aggression.

## Introduction

Over the last decades, there is clear progress in biosocial perspectives on the risk factors and origins of antisocial behavior (for an overview see Raine, 2013). The research on potential biological influences on aggression and delinquency ranges from behavior genetics, genetic interactions with social experiences to nutrition (Omega-3), hormonal factors and functional processes in the brain. An impaired connectivity between the amygdala and the dorsolateral prefrontal cortex seems to be particularly relevant for an adequate stress response and self-control in behavior (Glenn and Raine, 2008). In spite of the clear progress in biosocial research on aggression and crime, we must be aware that several studies contain small samples, cross-sectional data, extreme groups (e.g., psychopaths), and partially divergent results. As in other fields of research on antisocial behavior, more replicated and differentiated research is needed (Lösel, 2018; Farrington et al., 2019).

### Theoretical Background

An important field of research on biological risks for aggression and crime relates to the hypothalamic-pituitary-adrenal (HPA) axis that is a key system in the reaction to challenging and stressful situations. The steroid cortisol mobilizes physical resources under stress, potentiates fear and sensitizes for punishment (Van Goozen, 2005; Susman, 2006; Raine, 2013). Various theories aim to explain a relation between low adrenocortical activity and antisocial behavior. For example, low cortisol activity can go along with autonomous under-arousal (e.g., Eysenck, 1977) and a need for stimulation (e.g., Zuckerman, 1979; Van Goozen et al., 2007). This may lead to reduced harm-avoidance and reward dependence, enhanced fearlessness of adult psychopaths and callous-unemotional traits in youngsters (Hare, 1998; Raine, 2013; Frick et al., 2014). Whereas, these hypotheses primarily focus on biological dispositions, other approaches explain low cortisol reactions as a downregulation due to childhood adversity and stress (e.g., Bunea et al., 2017) or enhanced life strain (e.g., Agnew, 2014).

The theoretical bases of a relation between low HPA axis activity and antisocial behavior are similar to the research on low resting heart rate (RHR). Here, meta-analyses revealed significant relations of low RHR to antisocial behavior in both cross-sectional and longitudinal designs and for various forms of aggressive and delinquent behavior (Ortiz and Raine, 2004; Portnoy and Farrington, 2015). In the review of Portnoy and Farrington (2015) the mean effect of 114 studies on RHR and antisocial behavior was small (*d* = −0.20), but more robust than that on the relation between cortisol parameters and antisocial behavior. Here, a meta-analysis of Alink et al. (2008) on cortisol reactivity and externalizing behavior problems in children and adolescents found no significant mean effect (27 studies; *r* = −0.04) and a very small effect in studies on basal cortisol (72 studies; *r* = −0.05). In adolescent samples there was no significant relation. The mean effect in this meta-analysis on the relation between adrenocortical activity and antisocial behavior point to rather heterogeneous findings in sound studies. Some found the expected relation, and others not (e.g., Van Goozen et al., 2000; Snoek et al., 2004; Blair et al., 2005). Such differences in results may have been due to different samples, measurements, kind and intensity of behavioral problems. But the heterogeneity in findings seems not to be reduced since the meta-analysis of Alink et al. (2008). For example, Dietrich et al. (2013) found no significant relation between cortisol activity and antisocial behavior and Pascual-Sagastizabel et al. (2019) confirmed it only for boys but not for girls. Barrios et al. (2017) observed a small positive correlation between cortisol reactivity and externalizing child behavior problems at preschool age. These and other studies suggest more conceptual and empirical differentiation with regard to the relation between cortisol level and aggression. For example, lower cortisol levels seem to be particularly relevant in early onset, relatively persistently aggressive, and callous-unemotional youngsters (McBurnett et al., 2000; Loney et al., 2006; Stadler et al., 2011; Von Polier et al., 2013). This suggests that low cortisol stress reactivity relates more to instrumental aggression that goes along with emotional deficits (Blair, 2010).

### The Role of Protective Factors

Mainly small and partially inconsistent relations between biological variables and antisocial behavior are insofar plausible as numerous designs, measurements, sampling, and conceptual issues play a role. However, it is also relevant that the majority of research addresses biological factors only as *risk* factors and not as potential *protective factors* that can moderate the influence of risks and buffer against undesirable behavioral outcome. For example, Farrington (2020) showed that high RHR had a protective function in the prediction of conviction and antisocial personality characteristics when the individuals had experienced childhood risk factors. In comparison to risk oriented research, the investigation of protective factors and mechanisms of antisocial behavior is less developed (Lösel and Farrington, 2012). This is partially due to methodological issues because protective factors are not simply the “other side of the coin,” i.e., the opposite pole of a risk factor. The detection of buffering protective effects requires analyses of non-linear relationships, interaction effects or patterns of behavior in person-oriented analyses (Masten, 2013; Lösel and Bender, 2017).

In following these considerations, one should also take into account that a high cortisol level may function as a *protective* factor against antisocial development. On the personality trait level, this view is in line with findings on anxious and shy children who have enhanced levels of adrenocortical activity (Kagan, 1994, 2002; Dietrich et al., 2013). Anxiousness may reduce interest in adventures like criminal behavior and high anxiousness seems to protect against an early onset of delinquency (Tremblay et al., 1994; Farrington et al., 2016). However, in adolescence anxiousness may even become a risk factor for late onset or aggravation of offending (Loeber et al., 2008; Zara and Farrington, 2009). Some research suggests that an increase of cortisol in late childhood relates to an increase in aggressive behavior, but estradiol may have a buffering effect (Azurmendi et al., 2016).

### Complex Patterns of Influence

Obviously, the relation between adrenocortical activity and antisocial behavior is more complex than bivariate correlation coefficients can indicate. Dabbs et al. (1991) suggested that not the cortisol level alone but its interaction with testosterone is more relevant. This led to the dual-hormone hypothesis in the study of social aggression (e.g., Montoya et al., 2012). However, a recent meta-analysis showed only marginal support for the dual-hormone hypothesis for risk taking, aggression, dominance, and psychopathy (Dekkers et al., 2019). Other physiological factors have also been investigated as potential moderators (e.g., neuropeptides; Vaeroy et al., 2019). Psychological factors may also play a role in the relation between cortisol and aggression. For example, Pascual-Sagastizabel et al. (2019) found an interaction between cortisol level and empathy. However, empathy is a multidimensional construct whose relevance for antisocial behavior depends on various psychological and social conditions (e.g., Lösel, 2020). As in other fields of biological research on aggression and delinquency interactions with social factors are also important (e.g., Rutter et al., 2006). For example, Michels et al. (2012b) found a correlation between enhanced cortisol reaction and experience of family stressors in children. Jaffee et al. (2015) reported an interactive effect of exposure to stressful family experiences and children's cortisol in middle childhood. Barrios et al. (2017) showed that parental hostility was associated with externalizing child behavior symptoms at age three, and a combination with cortisol reactivity had a small relation to increased behavior problems 3 years later. Saxbe et al. (2012) found similar results in a cross-sectional study of adolescents.

Beyond the research on cortisol, studies have shown that high family stress, inappropriate parenting, economic deprivation, and other risk factors significantly predicted antisocial child behavior (for an overview see Lösel and Bender, 2006). Although causal directions are not yet clear, hormonal reactions to early life stress may play a mediating role in the development of child aggression (Winiarski et al., 2018). Similarly, outcomes of an intervention in childhood were related to the cortisol response in families at risk (O'Neal et al., 2010). Meta-analytic findings on older individuals reported a mean correlation between the experience of stressors and enhanced cortisol reaction, but there was much variance between studies (see Chida and Steptoe, 2009). Some research has shown that poverty, as an indicator of stressful life circumstances, was related to basal cortisol levels, but the relationship vanished in older children (Lupien et al., 2001) or was curvilinear in adolescence (Marsman et al., 2012).

The assumption of complex patterns in the relation between cortisol and aggression is in accordance with basic concepts of protective factors and resilience. Depending on other factors, a variable may have a protective function against a specific undesirable behavioral outcome, but may even be a risk for another (Bender and Lösel, 1997; Lösel and Farrington, 2012; Lösel and Bender, 2017). Accordingly, research on protective mechanisms should not only study protective factors in isolation, but consider broader developmental influences (Masten, 2011). What may protect against externalizing problems could even enhance the risk of internalizing problems (Lösel and Bender, 2003). The strength-oriented (salutogenetic) perspective of protective factors should be integrated with the deficit-oriented (pathogenetic) perspective to increase the amount of explained outcome variance (Bender and Lösel, 1998).

### Aims and Hypotheses of the Present Study

The present study mainly bases on the theoretical concept of arousal and related findings of cortisol and personality research. Within this framework, we investigate the risk and/or protective role of morning adrenocortical activity for aggressive behavior in a prospective longitudinal design that followed boys from preschool to middle school age. The cortisol awakening response (CAR) contains a quick hormonal increase in the morning after the circadian decrease during night. The cortisol-increase shortly after waking up may indicate an activation of memory, orientation to time and space, and other cognitive orientations for the upcoming day that is due to biological dispositions and stress experiences. It seems to be a particularly reliable indicator of a habitual hormonal reaction in these processes (Clow et al., 2004; Fries et al., 2009; Michels et al., 2012a).

Our review of literature revealed that the results on the relationship between cortisol and anti-social behavior of children are complex and only partially consistent. Due to the mainly cross-sectional study designs and the influence of third variables, we do not yet propose a definite causal model but aim to contribute to it with our study. According to the arousal hypothesis (e.g., Eysenck, 1977), an enhanced cortisol level should correlate negatively with aggression although previous findings are inconsistent (e.g., Alink et al., 2008). Research also suggests that anxious individuals have an enhanced cortisol level (Kagan, 2002). On the other hand, there is some comorbidity between anxiety and aggression (Angold et al., 1999). Therefore, it can be assumed that anxiousness might play a role in the relation of cortisol and aggressive behavior. A third construct that seems to be relevant for a differentiated model of the aggression-cortisol relation is stress in the family. On the one hand, family stressors may enhance the cortisol level (e.g., Michels et al., 2012b), and on the other hand, research has shown that family stress positively correlates with antisocial child behavior (Lösel and Bender, 2003). Here both risk and protective processes seem to be relevant.

To investigate the more complex relations of the mentioned constructs we will carry out not only variable-oriented moderator analyses, but also person-oriented behavior pattern analyses. The person-oriented approach has been advocated by Magnusson and Allen (1983) and gained attention in research on personality development and psychopathology (e.g., Bergman and Magnusson, 1997; Bergman and Lundh, 2015). It should not be seen as an alternative to variable-oriented analyses but as a complement that sometimes can show group differences that may also be practically useful as, for example, ICD and DSM still adhere to taxonomic instead of dimensional diagnostic categories. Behavior pattern analyses focus on prototypical groups and not on single clinical cases. In previous studies on developmental psychopathology, we have successfully applied the person-oriented strategy as a meaningful complement to our variable-oriented research (e.g., Lösel and Stemmler, 2012b; Stemmler and Lösel, 2012).

In summary, the following hypotheses can be derived within the above theoretical, empirical, and methodological framework: (a) As previous findings are mixed, we assume a relation between the children's morning adrenocortical activity and aggressiveness but the direction is yet unclear. (b) Due to relatively consistent findings, we expect a positive correlation between the children's cortisol level in the morning and their anxious withdrawn behavior. (c) According to previous research there should be a negative relation between the children's experience of family stress and both their cortisol level and the behavioral outcomes of aggressiveness and anxiousness. (d) We expect more complex relations between cortisol level and children's aggressive and anxious behavior, for example buffering (protective) effects of a high cortisol, and differentiated behavior patterns in variable-oriented and person-oriented analyses.

## Method

### Sample

Our data stem from the Erlangen-Nuremberg Prevention and Development Study (ENDPS; Lösel et al., 2013). This is a combined prospective longitudinal and experimental project that started at preschool age with children from 609 families in the area of Erlangen and Nuremberg in Germany (Lösel and Stemmler, 2012a). In the present study, we concentrated on boys only because we aimed for comparable biological data with regard to gender. Some studies had shown that cortisol profile in young male and female children are somewhat different (e.g., Freitag et al., 2009). And as in other studies (e.g., Moffitt et al., 2001; Moretti and Odgers, 2002; Lösel and Stemmler, 2012a), girls exhibited very low levels of physical aggressive behavior so that the base rate in the girls' group at preschool age was too low for a meaningful analysis. The boys were 4–6 years old at the first time of measurement (Time 1 = T1). Their mean age at T1 was *M* = 4.99 (*SD* = 0.54) years. At T1 the kindergarten or preschool teachers assessed the children's aggressiveness and anxiousness (see below). At this time, we also gathered data on children's cortisol level as well as on family stressors and ended up with a sample of *N* = 150 boys for whom we had complete data in all study variables at T1. In addition, we could include ratings on the children's behavior from schoolteachers about 6 years later (Time 2 = T2). Overall, we had complete data sets from *n* = 121 boys at both measurement times. At T2 the boys mean age was *M* = 10.95 (*SD* = 0.57).

Dropout in longitudinal studies is more or less normal. In our study, less than a quarter of families dropped out from T1 to T2, however, the rate for incomplete data sets was larger. There were various reasons for the reduction in sample size: Some families could not be reached at T2 for mobility reasons, others decided not to participate again in the study, some families only refused to participate in biological measurements, and some did not complete the family-related questions at T1. In addition to these family-related reasons, there was a sample reduction due to kindergarten educators/preschool teachers at T1 and school teachers at T2 who did not provide complete data on the children's social behavior. As the sample size was moderate and the sources for missing data varied, we decided against a statistical imputation of missing cases (e.g., Kleinke et al., 2011) and worked with the most valid complete data sets.

### Procedure

All measurements at T1 and T2 were only conducted with children for whom we had prior written informed consent from the parents. Families could have withdrawn their consent at any time.^1^

The following measurements and instruments were used in the present study.

#### Adrenocortical Activity

According to standards in endocrinological research we applied a frequently used method to assess boys' adrenocortical activity in the morning when cortisol level is normally highest. Cortisol levels can easily be determined in the saliva and we used medically approved hygienic cotton rolls from Sarstedt, Germany (www.sarstedt.com), to collect saliva samples at Time 1. We applied a standard procedure for assessment of the endogenous cortisol stimulation (e.g., Kirschbaum and Hellhammer, 1989).

The first sample was taken immediately after waking up (morning/awakening cortisol; C_AW_) and the second one about 30 min later (C_INC_). The respective difference between both measurements indicates the morning peak of the endogenous reaction (Fries et al., 2009). Although repeated measurements across the day or even over several days would have been ideal, this was not possible because our study contained a large set of social and psychological assessments in which biological variables were only a small part. Therefore, we concentrated on the boys' cortisol awakening response collected by the two measurements on the day of our family visit which is shown to be an often used and reliable index of adrenocortical reactivity (see Pruessner et al., 1997; Schmidt-Reinwald et al., 1999). Both assessments of the child were conducted by the parents who got a detailed instruction on how to apply the cotton rolls. The locked plastic tubes with the dampened cotton rolls were then put in their fridge and later collected by our research team for freezing in our institute. The cotton rolls with the saliva samples were analyzed anonymously in the neurophysiological laboratory of Hellhammer and Kirschbaum at the University of Trier in Germany.

**Figure 1:**
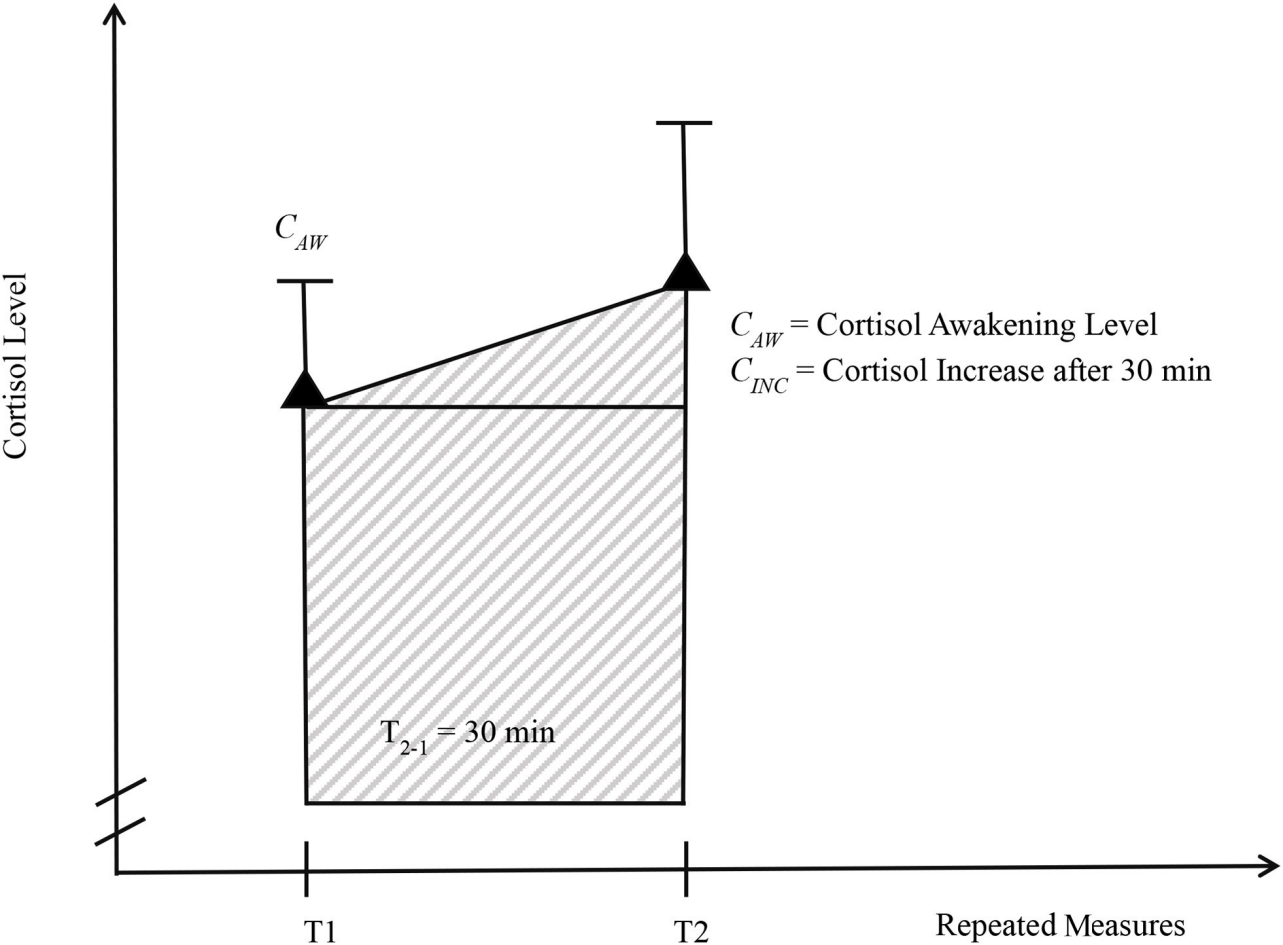
Visualization of the area under the curve with respect to the ground (*AUC*_*G*_) according to Pruessner et al. (2003, p. 918). *AUC*_*G*_ = Area under the curve with respect to the ground is represented by the shaded area in the rectangle and the triangle together.

For the analysis of boys' cortisol data, we computed the area under the curve with respect to the ground (*AUC*_*G*_). This is a frequently used method for repeated measurements in endocrinological research to assess the overall secretion of hormones over a specific time period and to estimate individual circadian changes. The *AUC*_*G*_ for the two measurements was calculated applying the formula suggested by Pruessner et al. (2003). Figure 1 visualizes this method with regard to our two measurements.

The resulting mean *AUC*_*G*_ score was *M* = 7.27 (*SD* = 2.70), whereby a higher score indicates an enhanced cortisol amount in the morning including the increase at the second measurement.

#### Children's Aggressive Behavior

At Time 1 the children's behavior problems in kindergarten were assessed by our German adaptation of the preschool version of the Social Behavior Questionnaire (SBQ) from Tremblay et al. (1987; see also Tremblay et al., 1992) that had shown good reliability (Lösel et al., 2006). The preschool version (PSBQ) as well as the school version (SBQ) is available for various informants. At Time 1 the children's social behavior was rated by the kindergarten teachers. Each item was rated on a three-point scale ranging from “*0”* = *never/not true* to “*2”* = *almost always/most of the time true*. In the present study, we used the subscale on “Physical Aggressiveness” (six items) that contains items on child behaviors like fighting, hitting, kicking, biting or bullying others or being cruel and mean. As this scale is relatively short, we added three items from the PSBQ scale on “Delinquency/Destruction” that indicate aggression against objects. The internal consistency of this enlarged scale was α = 0.87 (for descriptive statistics see Table 1).

**Table 1:** Descriptive statistics and correlations for study variables.

	***N***	***M***	***SD***	**1**	**2**	**3**	**4**	**5**	**6**
1. Cortisol (*AUC_G_)* T1	150	7.27	2.70	–	0.03	0.28^***^	−0.06	0.07	0.23^*^
2. Child aggressiveness T1	150	3.24	2.98		–	0.17^*^	−0.25^**^	0.37^***^	−0.03
3. Child anxiousness T1	150	2.63	2.64			–	−0.08	−0.05	0.20^*^
4. Family Stress T1	149	3.41	0.89				–	0.01	0.01
5. Child aggressiveness T2	121	1.36	2.58					–	0.15
6. Child anxiousness T2	121	2.46	2.71						–

*AUC_G_, Area under the curve with respect to the ground; T1, Time 1; T2, Time 2*.

* p < 0.05.

** p < 0.01.

*** *p < 0.001*.

To investigate potentially long-lasting consequences of the early behavior problems and the adrenocortical activity we assessed aggressiveness and anxiousness 6 years later (Time 2). Now the teacher version of the Social Behavior Questionnaire was used to assess the social behavior of children at school. The items are identical to the preschool version and schoolteachers rated the behavior of the child in the same three-point format as mentioned above. We again used the scale on “Physical Aggressiveness” plus the three items on destroying objects of the scale “Delinquency/Destruction.” The internal consistency was Cronbach's α = 0.89. It is important to mention that the schoolteachers did not know the results of the children's assessment at preschool age so that there was methodological blindness in this respect.

#### Children's Anxiousness

To measure the boys' anxiousness the scale on “Emotional Problems/Anxiousness” of the SBQ was used. It contains eight items (e.g., the child seems to be anxious, unhappy, sad, worried or tense). These symptoms were also rated on a three-point scale (from “*0”* = *never/not true* to “*2”* = *almost always/most of the time true*). At Time 1 the preschool version for kindergarten teachers and at Time 2 the teacher version for schoolteachers was applied. The internal consistency of the scale was α = 0.76 at T1 and α = 0.82 at T2.

#### Family Environment

We assessed the stress level of the children's families at Time 1 by an index that contained various objective variables. The basis was a standardized measure of socioeconomic status (SES; Geissler, 1994) which included weighted information on the parents' income, education, profession, and housing conditions. Although low SES is a well-replicated marker of accumulated family stress and health problems (e.g., Reiss et al., 2014), we added two objective psychological stressors of the family, namely having experienced parental divorce/separation and death of a close relative at Time 1 or close before. The resulting sum score of SES and the two dichotomized inverted family items indicated that children with a high score were living in a less stressful environment.

## Results

### Variable-Oriented Analyses

#### Bivariate Correlations

The means, standard deviations, and correlations for study variables at the two measurement times are shown in Table 1.

Similar to the mixed results in the literature, aggressiveness at Time 1 did not correlate significantly with the cortisol *AUC*_*G*_which also did not show predictive value for aggressiveness at Time 2. But as expected, at preschool age (Time 1) boys with higher anxiousness showed significantly higher cortisol *AUC*_*G*_ levels. Higher cortisol *AUC*_*G*_ also significantly predicted higher anxiousness in boys 6 years later (Time 2). There was also no significant correlation between enhanced cortisol *AUC*_*G*_ and the family stress at Time 1. When family stress was statistically controlled, the relation between cortisol and aggressiveness was nearly the same (*r* = −0.01, *ns*). Following Marsman et al. (2012), we also tested a potential curvilinear relation, but already an inspection of the scatter plot showed that this was not the case (neither for the stress index nor for SES alone).

As expected, boys' aggressiveness correlated significantly negative with our family stress indicator at Time 1. This showed that higher SES and less stressful life events were related to lower levels of aggressiveness, whereas anxiousness did not have a significant relation to it. The family stress index did not correlate with boys' behavior problems at Time 2 neither. Aggression at preschool age showed a significant correlation with anxiousness and significantly predicted aggression 6 years later. This was the highest correlation coefficient in Table 1. Boys' anxiousness at Time 1 significantly predicted anxiousness at Time 2, but less strongly.

#### Linear Regression Analyses

In a first step we included the variables in a cross-sectional regression analysis on aggressiveness at T1. The model was significant and explained eight percent of variance [*R* = 0.28, *F*_(3,145)_ = 4.21, *p* < 0.01]. Only family stress showed a significant contribution (*b* = −0.81, *se* = 0.27, *t* = −3.06, *p* < 0.01), whereas anxiousness (*b* = 0.15, *se* = 0.09, *t* = 1.60, *p* > 0.05) and *AUC*_*G*_ (*b* = −0.04, *se* = 0.09, *t* = −0.49, *p* > 0.05) did not. Longitudinally, the regression on aggressiveness at T2 with the four variables of T1 was also highly significant [*R* = 0.39, *F*_(4,116)_ = 5.34, *p* = 0.001], 16% of variance were explained but only aggression at T1 was a significant predictor (*b* = 0.35, *se* = 0.08, *t* = 4.49, *p* < 0.001). Anxiousness (*b* = −0.09, *se* = 0.09, *t* = −0.09, *p* > 0.05), family stress (*b* = 0.22, *se* = 0.26, *t* = 0.85, *p* > 0.05) and *AUC*_*G*_ (*b* = 0.10, *se* = 0.08, *t* = 1.16, *p* > 0.05) did not contribute to the explanation of variance.

#### Moderator Analyses

To test for more complex relations between the variables we carried out moderator analyses applying the PROCESS macro (version 3.5.3) by Hayes (2018). In the first analysis we included aggression as the dependent variable and the mean centered variables anxiousness and *AUC*_*G*_ of T1. This model was not significant [*R* = 0.19, *R*^2^ = 0.04, *F*_(3,146)_ = 1.89, *p* > 0.05]. None of the variables showed a significant contribution (anxiousness: *b* = 0.15, *se* = 0.10, *t* = 1.16, *p* > 0.05; *AUC*_*G*_: *b* = −0.03, *se* = 0.09, *t* = 1.23, *p* > 0.05; interaction term: *b* = 0.03, *se* = 0.03, *t* = 1.23, *p* > 0.05).

We also carried out a moderator analysis with *AUC*_*G*_ as the dependent variable and the mean centered variables anxiousness and aggression. The total effect of the model was highly significant [*R*^2^ = 0.09; *F*_(3,146)_ = 4.77, *p* < 0.001]. Anxiousness showed a significant contribution in the explanation of variance (*b* = 0.27, *se* = 0.08, *t* = 3.19, *p* < 0.01) whereas aggression not (*b* = −0.03, *se* = 0.07, *t* = −0.34, *p* > 0.05). The moderation between anxiousness and aggression did not reach significance [*R*^2^-change = 0.01, *F*_(1,146)_ = 1.45, *p* > 0.05] but the interaction term in itself correlated significantly with the cortisol level (*r* = 0.16, *p* < 0.05). A closer look at the moderator effect and its visualization showed that only for aggression values above the 50th percentile there was a significant correlation between anxiousness and *AUC*_*G*_ which indicated that children with both high aggressiveness and anxiousness scores had the highest cortisol levels and vice versa. But as the interaction term was in total not significant this could only be interpreted as a trend of a possible relation between the three variables.

We also found a negative correlation of a three-factor interaction of (high) *AUC*_*G*_, (low) family stress and (high) anxiousness with aggressiveness at T1 (*r* = −0.21, *p* < 0.01). However, since the total model was not significant and three-way interactions are often not replicated and difficult to interpret (Cohen et al., 2003; Dawson and Richter, 2006), we will not further discuss this finding.

The results of our variable-oriented analyses suggested more complex relations between the variables and indicated differentiated patterns of aggressive and anxious behavior in children. As sometimes in regression models, a strong effect of one variable may suppress a smaller but theoretically relevant other effect, we also conducted person-oriented analyses of prototypical patterns of behavior.

### Person-Oriented Analyses

For our person-oriented analyses we split the aggression and anxiety scales at Time 1 as close as possible at the respective medians and formed groups combining those above (+) and/or below (–) the median. The combination of the groups resulted in the following pattern: AG–AN– (*n* = 40), AG–AN+ (*n* = 35), AG+AN– (*n* = 35), and AG+AN+ (*n* = 40).

We compared the four groups in univariate ANOVAs. The means, standard deviations and *F*-values of study variables are presented in Table 2.

**Table 2:** Means (M) and standard deviations (SD) of the four groups in study variables.

	**AG–AN–^a^**		**AG–AN+^b^**		**AG+AN+^a^**		**AG+AN–^b^**			
	***M***	***SD***	***M***	***SD***	***M***	***SD***	***M***	***SD***	***F***	***df***
Aggressiveness T1	0.80	0.91	1.00	1.00	5.80	2.32	4.34	2.49	82.72^***^	3,146
Anxiousness T1	0.80	0.64	4.28	2.38	4.77	2.56	0.60	0.55	56.95^***^	3,146
Cortisol (*AUC_*G*_*) T1	6.75	2.57	7.82	2.20	8.04	2.78	6.45	2.93	3.28^*^	3,145
Family Stress T1	3.59	0.79	3.59	0.83	3.10	0.97	3.36	0.86	2.73^*^	3,145
Aggressiveness T2	0.40	0.77	0.57	0.95	2.00	3.01	2.58	3.70	6.02^**^	3,117
Anxiousness T2	1.86	1.92	3.54	3.30	2.33	2.92	2.29	2.71	2.17+	3,117

a n = 40,

b *n = 35. AUC_G_, Area under the curve with respect to the ground; T1, Time 1; T2, Time 2; AG, Aggressiveness; AN, Anxiousness; –, Scores below the Median; +, Scores above the Median*.

+ p < 0.10.

* p < 0.05.

** p < 0.01.

*** *p < 0.001*.

The results of the first two ANOVAs with boys' aggressiveness and anxiousness at Time 1 were expectable and only a validation of the group composition. There was a highly significant effect in aggressiveness with significant differences between the groups with aggressiveness scores above the median and the two groups with aggressiveness scores below it, namely between AG+AN+ and AG–AN– (mean difference *M*_*diff*_ = 5.00, *SEM* = 0.40, *p* < 0.001), between AG+AN+ and AG–AN+ (*M*_*diff*_ = 4.80, *SEM* = 0.42, *p* < 0.001), between AG+AN– and AG–AN– (*M*_*diff*_ = 4.54, *SEM* = 0.42, *p* < 0.001), and between AG+AN– and AG–AN+ (*M*_*diff*_ = 4.34, *SEM* = 0.43, *p* < 0.001).

The comparison of the groups in anxiousness was similarly highly significant with significant differences between the groups with anxiousness scores above the median and the groups with scores below it, namely between AG–AN+ and AG–AN– (*M*_*diff*_ = 3.48, *SEM* = 0.41, *p* < 0.001), between AG–AN+ and AG+AN– (*M*_*diff*_ = 3.68, *SEM* = 0.43, *p* < 0.001), between AG+AN+ and AG–AN– (*M*_*diff*_ = 3.97, *SEM* = 0.40, *p* < 0.001), and between AG+AN+ and AG+AN– (*M*_*diff*_ = 4.17, *SEM* = 0.41, *p* < 0.001).

With respect to the other study variables, there was an overall significant difference between the four groups in the cortisol measure *AUC*_*G*_. The AG+AN+ group had the highest mean and the AG+AN– the lowest (see Table 2). Between both groups there was a nearly significant difference in the *post hoc* comparison (*M*_*diff*_ = 1.59, *SEM* = 0.61, *p* = 0.08). The analysis of variance with the family stress index as the dependent variable did not show an overall significant difference between the four groups. But the two groups with aggressiveness scores above the median (AG+AN+ and AG+AN**–**) had the lowest mean stress score which means that they came by trend from families with a similarly high stress level compared to the two other groups who had less family adversities.

We also investigated whether the four groups formed at preschool age had different behavioral outcomes 6 years later. The results on teacher rated aggressiveness showed a clear effect in the analysis of variance (see Table 2). The groups with formerly high aggressiveness scores (AG+AN**–** and AG+AN+) had similarly high mean scores after 6 years. In contrast, the mean aggressiveness scores in the AG–AN– and AG–AN+ groups were much lower. The differential *post-hoc* contrasts showed significant differences between the groups AG+AN– and AG–AN– (*M*_*diff*_ = 2.18, *SEM* = 0.60, *p* < 0.01), between AG+AN– and AG–AN+ (*M*_*diff*_ = 2.00, *SEM* = 0.63, *p* < 0.05), and a nearly significant difference between AG+AN+ and AG–AN– (*M*_*diff*_ = 1.60, *SEM* = 0.62, *p* < 0.10).

The analysis of variance with teacher rated anxiousness 6 years later showed a weaker effect which was significant at *p* < 0.10 and accordingly there was no significant difference in the *post-hoc* contrasts between the four groups. However, the group AG**–**AN+ had the highest mean anxiousness score at Time 2, whereas the three other groups were clearly below that.

## Discussion

The present study aimed to investigate the relations between the amount of cortisol (after waking up and 30 min later) operationalized by the *AUC*_*G*_, aggressiveness, anxiousness and family stress within a sample of boys from a larger, prospective longitudinal study. It is a special strength of this study that we could assess adrenocortical activity already at preschool age and include a behavioral follow up of about 6 years. Such prospective longitudinal data are rare in the research on cortisol and children's antisocial behavior.

### Variable-Oriented Analyses

It was one of our hypotheses that a high morning cortisol level correlates with enhanced anxiousness what may be a protective mechanism against aggressive behavior of children. This assumption derived from various theories on the role of autonomous arousal in externalizing and internalizing behavior problems. As expected, we found a significant positive correlation between high adrenocortical activity and anxiousness at preschool age. There was even a significant correlation longitudinally. Anxiousness could be significantly predicted by the amount of cortisol in the morning (*AUC*_*G*_) that had been assessed 6 years before. This result fits to our hypothesis and suggests that the measurements were sufficiently reliable and valid. It is also in line with research that found enhanced adrenocortical activity in anxious and shy children (Kagan, 2002; Dietrich et al., 2013). As Loeber et al. (2008) and Farrington et al. (2016) have shown this personality characteristic can have a protective effect against the onset of antisocial behavior in development.

As previous findings are mixed with regard to the relation between aggression and cortisol, we expected a correlation but did not specify the direction. From a risk perspective, a low cortisol level may predict aggressive behavior. However, we did not find neither a cross-sectional nor a prospective longitudinal bivariate relationship between the amount of cortisol in the morning (*AUC*_*G*_) and aggressiveness. These findings are insofar in accordance with previous research as the relation between low cortisol and aggressiveness seems to be less robust than the relation between high cortisol reaction and anxiousness. Research has shown that the respective results are rather heterogenous (e.g., Van Goozen et al., 2000; Snoek et al., 2004; Blair et al., 2005; Alink et al., 2008; Dietrich et al., 2013; Pascual-Sagastizabel et al., 2019). Our findings underline those views in the literature that suggest no simple relation between aggression and cortisol, but recommend more differentiated interaction or pattern analyses. This is in accordance with the general research on protective factors against antisocial behavior (e.g., Lösel and Farrington, 2012; Lösel and Bender, 2017).

In our study, the cortisol level in the morning (*AUC*_*G*_) did not correlate significantly with the index of family stress. This supports the assumption that the morning cortisol reaction may indicate a more genuine biological characteristic than a reaction to later stressors during the day (Pruessner et al., 1997). As the cortisol level correlated with anxiousness, repeated social influences in the family may also have played role. However, as mentioned above, the results on the influence of family factors on cortisol levels are also mixed (e.g., Lupien et al., 2001; Marsman et al., 2012; Saxbe et al., 2012; Agnew, 2014; Bunea et al., 2017). Probably, both genetic dispositions and repeated family experiences are relevant for our findings. More replications on the relation between cortisol and social contexts are necessary.

If replicated, our results would have theoretical relevance for the broader topic of arousal and antisocial behavior. As mentioned in the introduction, meta-analyses found a negative correlation between resting heart rate (RHR) and antisocial behavior (e.g. Portnoy and Farrington, 2015). Our and other findings on the relation between high cortisol level and anxiousness suggest that it is not only the antisocial group of the respective samples that led to the negative correlation between low RHR and antisocial behavior, but also the proportion of the anxious/withdrawn individuals with high arousal and low antisociality. We are currently working on this hypothesis in a study on RHR and heart rate under stress.

Our multivariate variable-oriented analysis showed no significant influence of family stress in relation to cortisol but to aggressiveness at Time 1. This is in line with many studies on the development of aggression in children (see Lösel and Bender, 2006). Moderator analysis with regard to the cortisol level at T1 did not find a significant interaction effect of anxiousness with aggressiveness but a closer look at the nature of the moderation indicated, in contrast to the bivariate arousal hypothesis, that higher scores in aggressiveness were associated with a higher cortisol level, but only in cases of simultaneously enhanced anxiousness.

### Person-Oriented Analyses

The findings of our variable-oriented moderator analyses suggested interactions between cortisol, aggressiveness and anxiousness. This became more obvious in our person-oriented analysis: The group who was relatively high in both anxiousness and aggressiveness had the highest amount of cortisol in the morning (*AUC*_*G*_) and differed significantly from the group with low anxiousness and high aggression that showed the lowest one. This finding can be interpreted by concepts of different types of aggressive behavior, i.e., reactive vs. proactive aggression (Vitaro and Brendgen, 2012). Whereas the second type supports the assumption of a callous-unemotional disposition for aggression (e.g., Blair, 2010; Frick et al., 2014), the first one indicates a more emotional aggressive propensity. Our finding demonstrates the potential “double face” of the role of anxiousness in the development of antisocial behavior. On the one hand, it may protect against an antisocial pathway, on the other hand, it indicates a comorbidity that may act as an additional risk factor, e.g., when the youngsters get older (Loeber et al., 2008; Zara and Farrington, 2009). At later age, extreme cases in both subgroups may represent secondary vs. primary psychopathy and different underlying neurobiological processes (Blackburn, 1998; Vidal et al., 2010; Sethi et al., 2018). Our pattern analyses are insofar valid as the four groups at Time 1 also differed in aggressiveness 6 years later. The groups with low anxiousness and high aggressiveness and with both high anxiousness and aggressiveness at Time 1 were more aggressive at Time 2.

### Assessment of the Child Behavior

As mentioned above, the schoolteachers who rated the children's behavior had no knowledge about the assessments at preschool age. Ratings of child behavior by different informants in different contexts often show only small correlations (Lösel, 2002; Achenbach, 2006). Therefore, the significant variable- and person-oriented relations in our study endorse the reliability of the outcome data. In our variable-oriented analysis, the assessment of the children's aggressiveness at preschool age correlated significantly with the ratings from the schoolteachers 6 years later. With *r* = 0.37 the correlation is similar to what we found in a much larger sample (*r* = 0.35; Wallner et al., 2018). The above correlations for anxiousness by the different informants and in different contexts were also significant, but lower. The development of child behavior is often flexible and the distinction between an early starting life-course-persistent vs. an adolescence-limited pathway (Moffitt, 1993) is only one important differentiation in the context of others (e.g., Jennings and Reingle, 2012). However, externalizing problems like aggressiveness are often more stable than internalizing problems like anxiousness and seem to be a particularly important marker of an enhanced risk for behavioral and social problems in later life (e.g., Robins and Price, 1991; Bender and Lösel, 2011).

## Limitations

Although most of our findings are theoretically plausible, there are various limitations. First, there are limits concerning the cortisol measurement. In our broad gathering of social and psychological data in the families, we could only carry out two cortisol measurements. In addition, the application of the cotton roles to collect saliva samples did not take place under controlled laboratory conditions. Although we had given detailed instructions to the parents, we cannot be sure whether everybody followed these exactly. Hormone measurements are vulnerable for errors and there could have been some unreliability in our cortisol assessment. Repeated measurements in long-term studies show significant, but moderate stability (Platje et al., 2013). As we excluded extreme outliers and found significant relations between anxiousness and cortisol in both the cross-sectional and longitudinal design, our assessments seemed to be *relatively* reliable.

A second limitation concerns our sample. Compared to some other studies on cortisol reaction in children our sample was not small, but as not all parents participated in the biological part of our study, we cannot determine how representative our final data set is. In addition, the area of Erlangen-Nuremberg in Bavaria has a relatively affluent population so that extreme poverty and related stress may not be as prevalent as in some more deprived urban areas in Germany. Perhaps this range restriction had an influence on some of our effect sizes, e.g., for the correlations with the family stress index.

A third limitation concerns the measurement of the children's behavior problems. We could not carry out a detailed clinical assessment but had to rely on ratings from the kindergarten teachers in early childhood and from the schoolteachers 6 years later. We cannot be sure whether these ratings were always valid, particularly with regard to the less visible internalizing problems of anxiousness and withdrawal. Although a clinical assessment would have been preferable, we can insofar trust our measures as we applied the Social Behavior Questionnaire (SBQ; Tremblay et al., 1992) that is a standardized reliable and frequently used instrument. Our child behavior assessments showed also significant correlations between Time 1 and Time 2, although the SBQ data stem from different informants, in different contexts, and with 6 years of time difference. As mentioned above, under these conditions our significant longitudinal correlations are satisfactory.

## Conclusions

Overall, our study suggests that adrenocortical activity can play a role in the risk for and protection against aggressive development in children. However, it should not be studied and interpreted in isolation, but requires analyses in the context of other personality and social variables. Our study supports some theoretically meaningful differentiations. In particular, potentially protective or risk effects of adrenocortical activity seem to depend on the personality factor of anxiousness that is partially associated with the cortisol response. However, as in other fields of criminology and developmental psychopathology we need more replications (Lösel, 2018; Farrington et al., 2019).

More generally, our study on adrenocortical activity in children endorses the view that the research on biological risk and protective factors requires consideration of complex patterns of biological, personality, and social influences. Our results showed that single variables or constructs have a meaningful, but rather moderate impact. It is important for both research and practice to consider complex patterns and bring together biological, psychological, and social perspectives. There is still some controversy about the relevance of biological factors in criminology (see Raine, 2013). Perhaps, our article can contribute to a further reduction of such controversies that were formerly virulent between leading scholars like Sheldon and Eleanor Glueck vs. Edwin Sutherland. However, from a closer historical look they already contained substantial concordance (Laub and Sampson, 1991).

## Data Availability Statement

The raw data supporting the conclusions of this article will be made available by the authors, without undue reservation.

## Ethics Statement

Ethical review and approval was not required for the study on human participants in accordance with the local legislation and institutional requirements. Written informed consent to participate in this study was provided by the participants' legal guardian/next of kin.

## Author Contributions

All authors listed have made a substantial, direct and intellectual contribution to the work, and approved it for publication.

## Conflict of Interest

The authors declare that the research was conducted in the absence of any commercial or financial relationships that could be construed as a potential conflict of interest.

## Footnotes

^1^The instruments and procedures of our project were approved by the German Federal Ministry of Family Affairs and the Friedrich-Alexander-Universität Erlangen-Nürnberg.
